# Hydrothermal Synthesis of Various Shape-Controlled Europium Hydroxides

**DOI:** 10.3390/nano11020529

**Published:** 2021-02-19

**Authors:** Hongjuan Zheng, Kongjun Zhu, Ayumu Onda, Kazumichi Yanagisawa

**Affiliations:** 1 State Key Laboratory of Mechanics and Control of Mechanical Structures, Nanjing University of Aeronautics and Astronautics, Nanjing 210016, China; zhenghj2012@126.com (H.Z.); kjzhu@nuaa.edu.cn (K.Z.); 2 Research Laboratory of Hydrothermal Chemistry, Faculty of Science, Kochi University, Kochi 780-8520, Japan; aonda@kochi-u.ac.jp

**Keywords:** rare earth, europium hydroxides, hydrothermal, morphology control, nanoparticles

## Abstract

Eu(OH)_3_ with various shape-controlled morphologies and size, such as plate, rod, tube, prism and nanoparticles was successfully synthesized through simple hydrothermal reactions. The products were characterized by XRD (X-Ray Powder Diffraction), FE-SEM (Field Emission- Scanning Electron Microscopy) and TG (Thermogravimetry). The influence of the initial pH value of the starting solution and reaction temperature on the crystalline phase and morphology of the hydrothermal products was investigated. A possible formation process to control morphologies and size of europium products by changing the hydrothermal temperature and initial pH value of the starting solution was proposed.

## 1. Introduction

Rare earth (RE) compounds have drawn continuous research attention for many years because of their unique optical, magnetic, electric, and catalytic properties resulting from their unique 4f-electron configuration. The several frequently used RE compounds currently include RE oxides [1,2,3], hydroxides [4,5,6], fluorides [7,8,9], phosphates [10,11], and so on. Certainly, RE hydroxides are of great importance because RE oxides can be directly formed through dehydration of their corresponding hydroxides, and RE sulfides can be directly obtained through sulfuration of the hydroxides. The hydroxyl group may also act as active sites for grafting other organic or inorganic functional groups [12]. Therefore, RE hydroxides are an important intermediate to synthesize oxides, sulfides, or other functional materials.

The properties of RE compounds depend strongly on their morphologies. Many kinds of morphological RE compounds depending on their inherent anisotropy have been explored in the recent decades, such as one-dimensional (1D) nanostructures (nanowires, nanorods, nanotubes), two-dimensional (2D) (nanosheet, nanobelts) and three-dimensional (3D) architectures (flower-like [13], spindle-like [14], hierarchical architectures [15]). In order to achieve the specific properties for their further applications, the selection of well-defined synthetic methods is required.

Among all the RE ions, Eu^3+^ can be effectively activated by ultraviolet rays or cathode rays and emits high purity red light because of its unique 4f^6^ configuration [16]. The major emission band of Eu^3+^ is centered near 611 nm (red), which is one of the primary colors [17]. Therefore, Eu^3+^ is a good activator with sharp and intense luminescence in the red region of the visible spectrum. Monodisperse hexagonal Eu(OH)_3_ submicrospindles with a diameter of 80−200 nm and a length of 500−900 nm have been synthesized in a large scale via a facile aqueous solution route by Xu et al. [18]. The morphology of Eu_2_O_3_ obtained by the calcination of Eu(OH)_3_ submicrospindles maintains the same morphology as Eu(OH)_3_. The similar method of annealing the Eu(OH)_3_ precursor to obtain Eu_2_O_3_ with the same morphology as the former have been reported by others in succession in recent years [19,20,21]. Xu et al. [22] obtained the monodisperse and well-defined 1D rare earth fluorides (β-NaREF_4_) (RE = Y, Sm, Eu, Gd, Tb, Dy, and Ho) nanowires/nanorods using RE(OH)_3_ as precursors via a facile hydrothermal route and characterized their photoluminescence properties. Among them, the diameter and length of Eu(OH)_3_ nanowires are about 10−20 nm and 0.1–0.2 μm, and the final β-NaEuF_4_ samples inherit their Eu(OH)_3_′s morphology by this conversion process. Zhang et al. [23] synthesized the biocompatible Eu(OH)_3_ nanoclusters composed of approximately 5 nm nanoparticles with a modified microwave-assisted hydrothermal method and showed the as-synthesized Eu(OH)_3_ nanoclusters exhibited excellent physiological stability and biocompatibility both in vitro and in vivo, and possessed considerable pro-proliferative activities in human umbilical vein endothelial cells. This study developed the application of Eu(OH)_3_ to biofunctional nanomaterials.

Because of the unique nature of RE ions, the assembly of Eu complexes offers great challenges and opportunities in terms of controlling fascinating frameworks and specific properties. To date, various approaches have been used to prepare Eu(OH)_3_ particles, such as homogeneous precipitation [24], solvothermal [25], microwave [23], and the hydrothermal method [21], and so on. The hydrothermal method has drawn tremendous attention owing to its advantages, such as simplicity, low energy consumption, environmental friendliness, and well-defined product morphology.

Eu(OH)_3_ will be continuously studied for development of their various applications. At this point, it is particularly important to exploit unique products with a variety of morphologies. In this study, we proposed a simple hydrothermal route for the synthesis of Eu(OH)_3_ nanoparticles with various controlled morphologies. The influence of the pH value of the starting solutions and reaction temperature on the crystallized phase, particle size, and morphology of europium compounds was systematically investigated. In addition, the mechanism of morphology evolution under hydrothermal conditions was proposed.

## 2. Experimental

### 2.1. Raw Materials

The starting materials, europium oxide (Eu_2_O_3_) (99.9%), nitric acid (HNO_3_) (60%) and ammonia (NH_3_·H_2_O) (28%), were received from Wako Pure Chemical Industries, Ltd. (Osaka, Japan). All chemicals were used as received without further purification.

### 2.2. Preparation of Eu(OH)_3_ Powders

In a typical synthesis process, 0.45 g of Eu_2_O_3_ was dissolved in 6ml of 3.0 M HNO_3_ solution through hydrothermal treatment at 120 °C for 2 h. Then, NH_3_·H_2_O was added to adjust the pH of the solution to a designated value for getting the precipitates under vigorous agitation. When NH_3_·H_2_O was added to the Eu solutions, the transparent solutions were changed to opaque colloidal solutions consisting of amorphous particles. The range of pH value was between 7 and 12 (the maximum pH value adjusted with a concentrated ammonia solution is about 12). The final volume of the resulting solution was adjusted to reach 15 mL. The as-obtained colloidal solution was immediately transferred into a 25 mL Teflon-lined autoclave (homemade, Kochi, Japan), followed by hydrothermal treatment at temperatures from 80 to 220 °C for 24 h. After cooling, the received white precipitate was collected by a centrifuge, washed with distilled water several times and dried at 80 °C overnight.

### 2.3. Characterization

The as-prepared powders were characterized by X-ray powder diffraction (XRD) to determine the crystal structure under a Rigaku RTP-300RC X-ray diffractometer (Tokyo, Japan) with Cu Kα radiate on (λ = 1.5418 Å). Specific scan parameters were a tube voltage of 40 kV and tube current of 20 mA. The patterns were collected in the range of 5 to 70° with a 0.02° step and scanning speed of 4°/min. Field emission scanning electron microscopic (FE-SEM) observation was carried out on a JEOL JSM-6500F instrument (Tokyo, Japan) at 15 kV to collect the structure information of the powders. Thermogravimetry- Differential Thermal Analysis (TG-DTA) study was performed using a TG/DTA System (Mac Science Co. Tokyo, Japan, TG-DTA2020S) in air. The temperature was raised from room temperature to 900 °C at a heating rate of 10 °C/min, and held at 900 °C for 10 min.

## 3. Results and Discussion

### 3.1. Crystalline Phases

**Figure 1:**
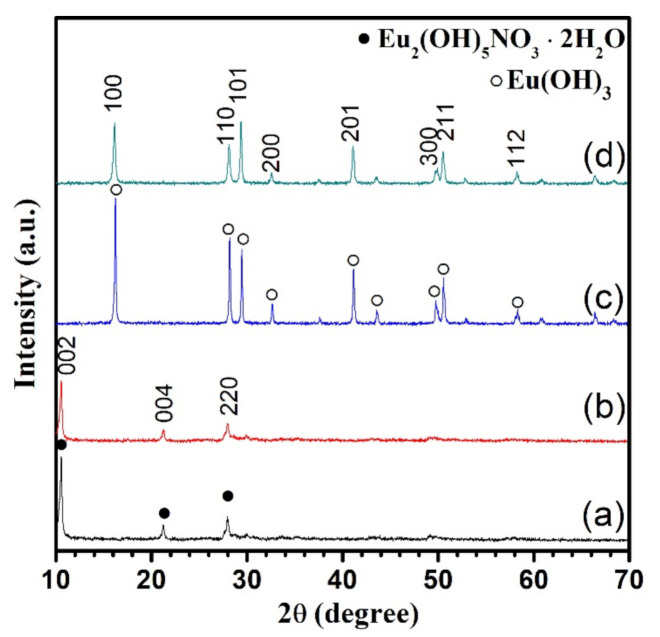
XRD patterns of the products obtained at 80 °C for 24 h as a function of the pH value of the starting solution. The pH value used is 7.34 (**a**), 9.24 (**b**), 10.82 (**c**), 12 (**d**).

The crystal structure of the hydrothermally synthesized europium products was characterized by XRD, as shown in Figure 1. First, the lowest reaction temperature was fixed to 80 °C, and the pH value of the starting solution was increased from 7 to 12. The positions of diffraction peaks of the products obtained with the pH value of 7.34 and 9.24 are the same. The diffraction patterns exhibit a series of strong (001) and sharp (220) diffractions, suggesting that all the peaks can be well indexed to a layered phase [26], as shown in Figure 1a,b. These diffraction peaks show exactly the same patterns with Eu-doped Y_2_(OH)_5_NO_3_·2H_2_O according to the result reported by Zhang et al. [27] and Wu et al. [28] Therefore, the products can be temporarily identified as the pure phase of Eu_2_(OH)_5_NO_3_·2H_2_O. As the pH value was increased to 10.82, the pure phase of Eu(OH)_3_ was successfully formed, as shown in Figure 1c. The diffraction peaks matched the standard data of a hexagonal phase with a P63/m space group, according to the JCPDS Card No. 83-2305. When the pH value was higher than 12, the pure hexagonal phase of Eu(OH)_3_ was also synthesized, as shown in Figure 1d. It is important to note that the relative intensity ratio of (110) and (101) diffraction was changed with the increase in pH value of the starting solution, suggesting that the preferential crystal growth direction of two kinds of Eu(OH)_3_ products might be different. In other words, the crystals of these products might show different morphologies. The intensity of the main diffraction peak (100) of the product obtained in the solution with the highest pH value was lowest, which indicated that the crystallinity of Eu(OH)_3_ was decreased with the increase in pH value of the starting solution.

**Figure 2:**
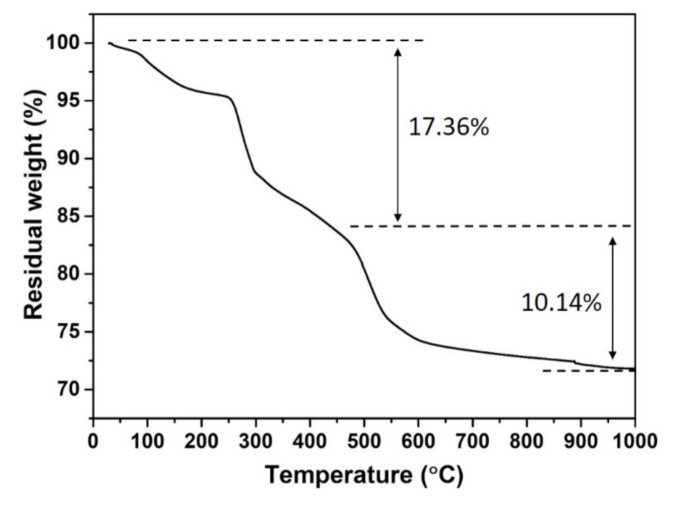
TG curves of the product obtained at 80 °C for 24 h in the solution with a pH value of 7.34.

Figure 2 shows the TG curve of the product synthesized at 80 °C for 24 h in the solution with a pH value of 7.34. It can be concluded that the weight loss of 17.36% below 450 °C corresponds to the evaporation of water and the release of OH species. The weight loss at temperatures higher than 450 °C is 10.14%, which associated with the release of NO species. The overall weight loss of 27.5% is consistent with the theoretical value of 27.7% of the transition from Eu_2_(OH)_5_NO_3_·2H_2_O to Eu_2_O_3_. The above analysis is consistent with the thermal decomposition of Y_2_(OH)_5_._14_(NO_3_)_0_._86_·H_2_O reported by Li et al. [2] Therefore, the product obtained in the solution with a pH value of 7.34 was proved to be Eu_2_(OH)_5_NO_3_·2H_2_O.

**Figure 3:**
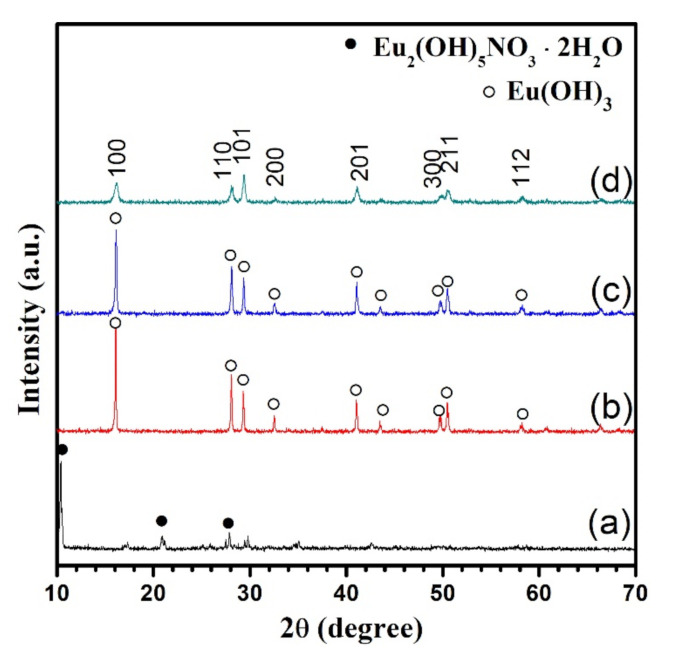
XRD patterns of the products obtained at 160 °C for 24 h as a function of the pH value of the starting solution. The pH value used is 7.26 (**a**), 8.99 (**b**), 10.48 (**c**), 12 (**d**).

To obtain a further understanding of the effect of the pH valve on the crystal forms of hydrothermally synthesized europium products, Figure 3 exhibits the XRD patterns of hydrothermally synthesized europium products at 160 °C for 24 h as a function of the pH value of the initial hydrothermal solution. According to the XRD patterns, when the pH value was 7.26, the diffraction peaks can be indexed to be the layered phase of Eu_2_(OH)_5_NO_3_·2H_2_O, as shown in Figure 3a. When the pH value was higher than 8.99, the pure hexagonal phase of Eu(OH)_3_ can be formed, as shown in Figure 3b–d. However, the crystallinity of Eu(OH)_3_ was gradually decreased with the increase in pH value of the starting solution, suggested by the decrease in the intensity of the main diffraction peak (100) of the product.

**Figure 4:**
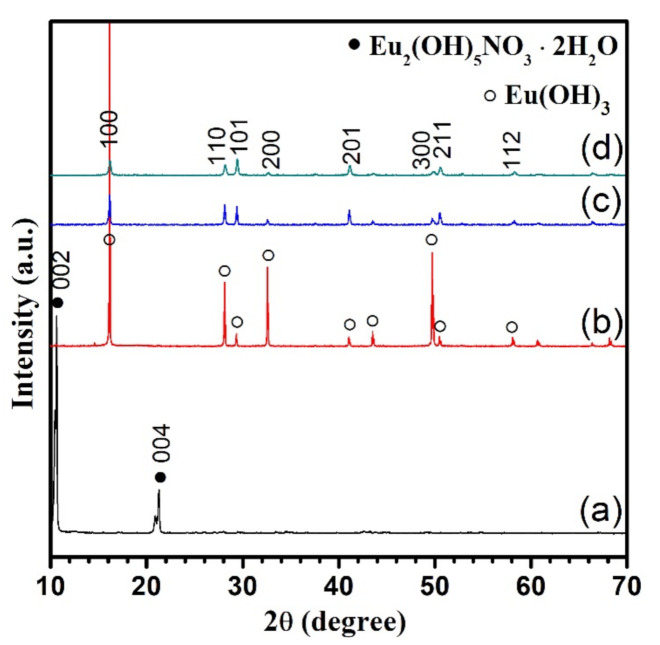
XRD patterns of the products obtained at 220 °C for 24 h as a function of the pH value of the starting solution. The pH value used is 7.63 (**a**), 9.15 (**b**), 10.90 (**c**), 12 (**d**).

Finally, the effect of the pH valve on the crystals form of hydrothermally synthesized europium products at high temperature of 220 °C was further investigated. The crystallized phases of the synthesized europium products were similar to the products obtained at 160 °C, as shown Figure 4. Unlike the Figure 1a,b and Figure 3a, the (220) diffraction of layered Eu_2_(OH)_5_NO_3_·2H_2_O crystals prepared at 220 °C were disappeared, which suggests that the crystals tend to grow oriented in the *c* axis direction. The crystallinity of Eu(OH)_3_ crystals synthesized in the solution with a pH value of 9.15 (Figure 4b) was obviously higher than in the solution with a pH value of 10.90 (Figure 4c) and 12 (Figure 4d) according to their intensity of diffractions.

### 3.2. Morphology

**Figure 5:**
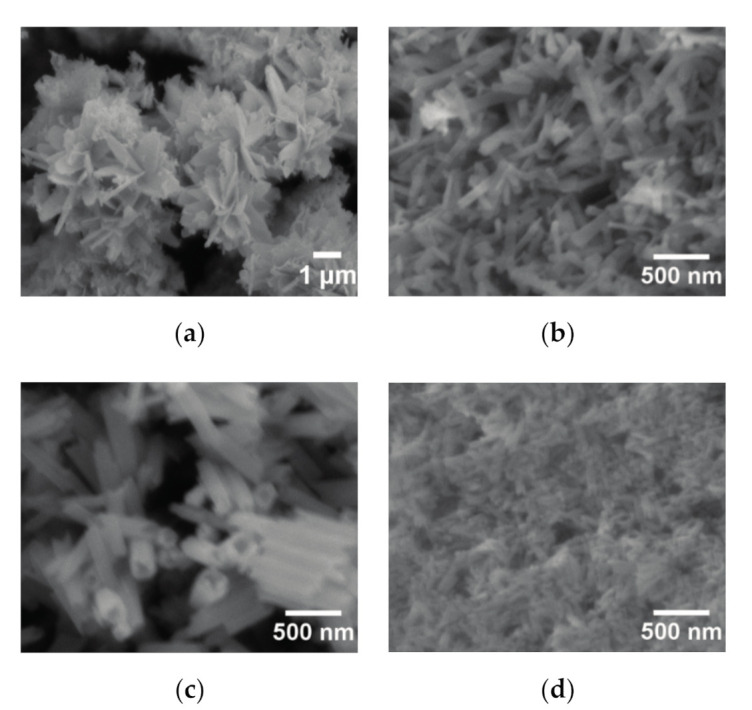
SEM images of the products obtained at 80 °C for 24 h as a function of the pH value of the starting solution. The pH value used is 7.34 (**a**), 9.24 (**b**), 10.82 (**c**), 12 (**d**).

In addition to the crystalline phases obtained, the pH value of the initial solutions and reaction temperature also have an impact on the morphology. The morphology of the products synthesized at 80 °C for 24 h was shown in Figure 5 as a function of the pH value of the starting solutions. When the pH of the starting solution was 7.34, the Eu_2_(OH)_5_NO_3_·2H_2_O crystals comprised flower-like agglomerates, which contained the ultrathin plate-like crystals with a size of above 1 μm, as shown in Figure 5a. As the pH was increased to 9.24, Eu_2_(OH)_5_NO_3_·2H_2_O crystals were composed of nanorods with a diameter of 100 nm and a length of 400–500 nm, as shown in Figure 5b. Eu(OH)_3_ crystals synthesized in the solution with a pH value of about 11 is shown in Figure 5c. Eu(OH)_3_ crystals consisted of nanotubes with an outer diameter of 200–300 nm, an inner diameter of 80–100 nm, and a length of 500 nm. Figure 5d reveals that the fine Eu(OH)_3_ nanorods with diameter of 30–40 nm and length of 100 nm were formed in the solution with a pH value of about 12.

**Figure 6:**
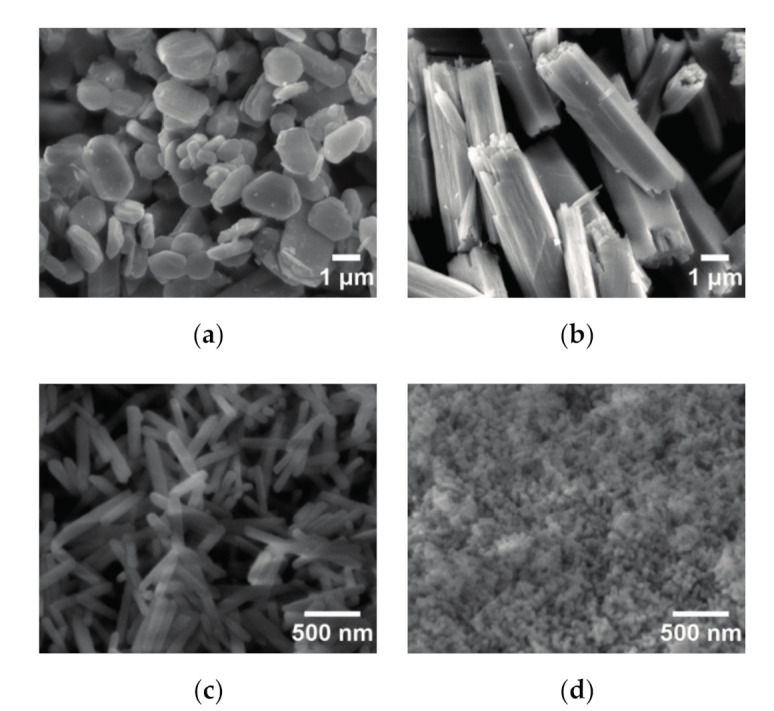
SEM images of the products obtained at 160 °C for 24 h as a function of the pH value of the starting solution. The pH value used is 7.26 (**a**), 8.99 (**b**), 10.48 (**c**), 12 (**d**).

Figure 6 shows SEM images of the products synthesized obtained at 160 °C for 24 h as a function of pH value of the starting solution. Eu_2_(OH)_5_NO_3_·2H_2_O crystals obtained in the starting solution with pH 7.26 were composed of small disk-like crystals with a diameter of 1.5–2 μm, as shown in Figure 6a. This disk-like morphology was very close to Y_2_(OH)_5_._14_(NO_3_)_0_._86_·H_2_O in the shape of a sheet that was reported by Li, et al. [2] As the pH was increased to 8.99, Eu(OH)_3_ crystals showed micro-cylinders with a diameter of 1–2 μm and a length up to 6 μm, as shown in Figure 6b. When the pH was changed to 10.48, the synthesized Eu(OH)_3_ crystals showed the uniform nanorods with a diameter of 100 nm and a length of 400–500 nm, as shown in Figure 6c. Similar nanorod-like Eu(OH)_3_ crystals with a width of 50–150 nm and a length of 500 nm–2 μm were prepared by adding ammonia by Kang et al. [21] However, they neither gave the specific pH of the starting solutions nor the added amount of ammonia. The fine Eu(OH)_3_ nanoparticles with a diameter less than 50 nm were obtained in the solution with a pH value of about 12, as shown in Figure 6d.

**Figure 7:**
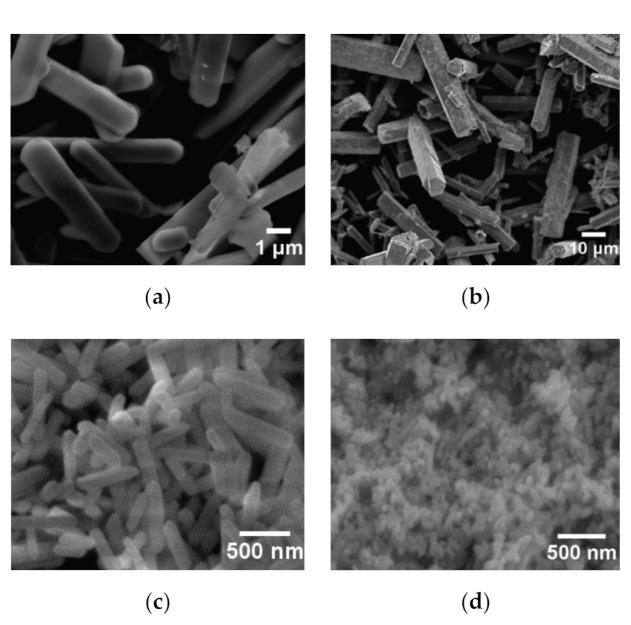
SEM images of the products obtained at 220 °C for 24 h as a function of the pH value of the starting solution. The pH value used is 7.63 (**a**), 9.15 (**b**), 10.90 (**c**), 12 (**d**).

Figure 7 shows SEM images of the products obtained at 220 °C for 24 h as a function of the pH value of the starting solution. When the pH of the starting solution was 7.63, the plate-like Eu_2_(OH)_5_NO_3_·2H_2_O crystals were changed to a long board shape with an average aspect ratio of five, as shown in Figure 7a, which means that the crystals grew along the *c* axis direction. These results were consistent with those of their XRD patterns. The hexagonal prism Eu(OH)_3_ crystals with a diameter of 8–10 μm and a length of 30–40 μm (Figure 7b) were obtained when the pH was changed to 9.15. Ji et al. [29] obtained the short hexagonal nano-prism Eu(OH)_3_ at 120 °C and pH 8.8 by adjusting it with a NaOH aqueous solution. When the precursor pH was increased from 8.8 to 9.5 at the same temperature, the hexagonal prisms became more slender and their aspect ratio changed from 1.1 to 2.1 [29]. Thus, smaller and shorter hexagonal prisms could be synthesized at a lower temperature when ammonia was replaced by NaOH. When the pH was further increased to 10.90, Eu(OH)_3_ crystals composed of nanorods with a diameter of 150 nm and a length of 700 nm were synthesized, as shown in Figure 7c. The fine Eu(OH)_3_ nanoparticles with a diameter less than 50 nm were obtained in the solution with a pH value of about 12, as shown in Figure 7d.

**Figure 8:**
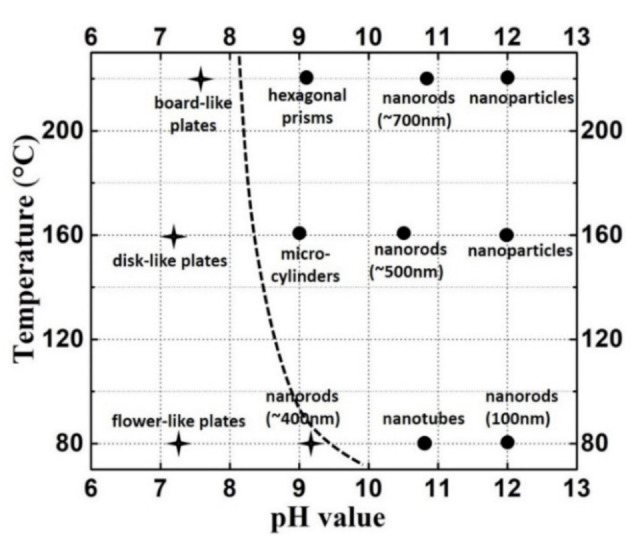
Morphology distribution in a temperature–pH diagram. (Closed circle: Eu(OH)_3_; star: Eu_2_(OH)_5_NO_3_·2H_2_O).

In this study, two crystalline phases, Eu(OH)_3_ and Eu_2_(OH)_5_NO_3_·2H_2_O, were obtained as a single phase depending on reaction conditions, and they are shown in Figure 8 with their characteristic morphologies. The left and right parts of the dashed line represent different phases, i.e., Eu(OH)_3_ (marked with a circle) on the right and Eu_2_(OH)_5_NO_3_·2H_2_O (marked with a star) on the left. To a certain degree, the dashed line represents the boundary of the two phases. A proposed formation process seems to be responsible for the observed development of the morphologies of Eu_2_(OH)_5_NO_3_·2H_2_O and Eu(OH)_3_ crystals synthesized with various pH values and temperatures. The presence of NO_3_^−^ and the concentration of OH^−^ are the essential factors for the formation of layered Eu_2_(OH)_5_NO_3_·2H_2_O compounds. Layered Eu_2_(OH)_5_NO_3_·2H_2_O crystals were easily formed in the solution with low pH of 7 and in the solution with a pH of 9 at a low temperature. When the temperature increased in the solution with pH 9, Eu(OH)_3_ was formed.

When the reaction temperature increased, Eu_2_(OH)_5_NO_3_·2H_2_O crystals grew larger in the solution with a pH value of about 7. This can be attributed to the Ostwald ripening process under hydrothermal conditions. The reaction temperature plays a key role in the dissolution of smaller grains and the growth of larger grains [30], as observed in Figure 5a and Figure 6a. A higher temperature of 220 °C not only results in the growth of grains, but also leads to the directional growth of grains along the *c* axis direction.

In the solution with pH 9, Eu_2_(OH)_5_NO_3_·2H_2_O crystallized at 80 °C but Eu(OH)_3_ crystallized at 160 °C. Eu(OH)_3_ is a stable phase at a higher temperature than Eu_2_(OH)_5_NO_3_·2H_2_O. The Eu(OH)_3_ tubes were obtained only by the experiment at 80 °C with pH 11. Eu_2_(OH)_5_NO_3_·2H_2_O precursor may be formed in the solution with pH 11 at lower temperature than 80 °C and it transforms to Eu(OH)_3_ quickly while heating to 80 °C. Nanotubes of Eu(OH)_3_ might be formed by rapid preferential growth along the circumferential edges in the c axis direction, similar to the formation of Y(OH)_3_ nanotubes [2]. At 220 °C, Eu(OH)_3_ crystals grew larger than at 160 °C. In the solution with pH 9, the temperature is still the main factor affecting the morphology and size of the grains. A high temperature promoted more quickly the dissolution of the smaller grains, and the larger grains grew up in order to reduce the surface energy. [31]

The size of Eu(OH)_3_ grains obtained in the solution with pH 11 were small. That might be attributed to the increase in pH value which leads to the decrease in the solubility of Eu(OH)_3_ precursors. However, Eu(OH)_3_ crystals grew larger with the increase in reaction temperature by the Ostwald ripening process. A large amount of ammonia was added to the solution to obtain the solution with pH 12. The solubility of hydroxides must be very low in such a solution with a high pH value. Many Eu(OH)_3_ crystal nucleus were rapidly formed when the starting solution was prepared, but they could not grow larger by the Ostwald ripening process due to the low solubility even at a high temperature. Thus the size of Eu(OH)_3_ crystals did not change, even at the highest temperature. Based on this mechanism discussed above, Eu(OH)_3_ crystals with more different morphologies, such as micro-cylinders, hexagonal prisms, nanotubes, nanorods and nanoparticles, are synthesized by adjusting the pH with ammonia and the temperature, even without the addition of surfactants [32].

## 4. Conclusions

The formation conditions of Eu(OH)_3_ crystals with controlled morphology and size were systematically investigated through simple hydrothermal reactions by adjusting the pH value of the starting solution and reaction temperature. When the pH value of the starting solution was about 7, Eu_2_(OH)_5_NO_3_·2H_2_O crystals rather than Eu(OH)_3_, with the flower-like agglomerates, disk-like plates and long boards, were obtained at the reaction temperature of 80 °C, 160 °C and 220 °C, respectively. In order to obtain Eu(OH)_3_, it is necessary to further increase the reaction temperature and pH value of the starting solutions. The characteristic morphology of Eu(OH)_3_ crystals such as micro-cylinders and hexagonal prisms could be formed in the solution with pH 9 at 160 °C and 220 °C, respectively. Nanotube and nanorod Eu(OH)_3_ with different lengths were formed at different temperatures in the solution with pH 11. Nanoparticles were obtained at above 160 °C in the solution with pH 12, due to the decrease in the solubility of Eu(OH)_3_ precursors in high pH solutions. The method utilized in this study to fabricate Eu(OH)_3_ crystals could be extended to synthesize the other RE hydroxides with tunable morphologies by simply adjusting the pH value and reaction temperature.

## Data Availability

The data presented in this study are available in this article.
